# Gene therapy for inborn errors of immunity: past progress, current status and future directions

**DOI:** 10.20517/rdodj.2025.42

**Published:** 2025-10-14

**Authors:** Robert Torrance, Kate Orf, Thomas A Fox

**Affiliations:** 1 UCL Institute of Immunity and Transplantation, UCL, London NW3 2PP, United Kingdom; 2 Department of Haematology, University College London Hospitals NHS Foundation Trust, London NW1 2PG, United Kingdom

**Keywords:** Gene therapy, inborn errors of immunity, primary immunodeficiency

## Abstract

Inborn errors of immunity (IEIs), also known as primary immunodeficiencies, are a group of rare inherited disorders that affect the immune system. They result in severe, opportunistic infections, severe autoimmune manifestations and a predisposition to malignancy. The only curative treatment for many years has been allogenic haematopoietic stem cell transplantation (alloHSCT). However, this requires the availability of a suitable donor and has risks of morbidity and mortality. Autologous gene therapy (GT) abrogates the immunological complications of alloHSCT and uses the patient’s own cells, removing the need for a donor. Preclinical proof-of-concept and clinical trials in humans have demonstrated that GT is safe and effective and can be used to correct a variety of IEIs. In this review, we outline the progress in developing GT for IEIs over the last four decades. We describe the gene editing technologies available to correct IEIs and their current applications. We also examine advances in GT development, the challenges to its application, and discuss future developments in the field, including emerging *in vivo* approaches.

## Introduction

Inborn errors of immunity (IEIs) are a group of rare inherited disorders that affect the immune system. Over 550 have been described to date^[1,2]^. Whilst individually rare, collectively IEIs affect approximately 1 in every 1,200 individuals^[2]^. In addition to increased susceptibility to infection, IEIs may present with autoimmunity, autoinflammation, and malignancy. Some IEIs, such as severe combined immunodeficiencies (SCID), require definitive treatment [e.g., allogeneic haematopoietic stem cell transplantation (alloHSCT) or gene therapy] early in life to prevent death from catastrophic infection. Non-SCID IEIs typically have more heterogenous clinical features both within and between individual diagnoses, with optimal treatment strategies varying depending on clinical presentation. Patients with limited clinical manifestations may be managed using targeted therapies or supportive care, whilst more severe disease requires alloHSCT.

For the majority of IEIs, alloHSCT is the only curative therapy, and, in many cases, is now the standard of care if a matched donor is available^[3]^. Since the first alloHSCT for IEIs more than 50 years ago, advances have been made in understanding the natural disease course of many indications^[4,5]^. This has enabled the publication of clear guidelines regarding when and how transplant should take place^[3]^. For example, the risk/benefit of alloHSCT is clear in diseases such as SCID, where without transplant the disease is universally fatal^[6]^. With current transplant practices, the survival rate is close to 95% in SCID patients transplanted under 3 months of age. Outcomes are expected to improve further with the expansion of universal newborn screening which will enable infants to undergo alloHSCT prior to the development of any infectious complications^[7,8]^.

However, decisions about when to proceed with alloHSCT in rarer IEIs are challenging, as the natural course of the disease is less certain^[9]^. Risks of alloHSCT in many patients with IEIs are further increased due to infectious complications and/or significant organ dysfunction prior to or peri-transplant which increases the risks of transplant-related mortality^[10]^. Furthermore, the risks of graft-versus-host disease (GVHD), poor graft function or graft failure are increased in some IEIs, particularly those with uncontrolled autoinflammation and autoimmunity prior to transplant^[10]^. Other longer-term risks following transplant are well characterized and include endocrinopathies, chronic GVHD, autoimmune conditions, secondary malignancies and impaired growth^[11]^.

**Figure 1:**
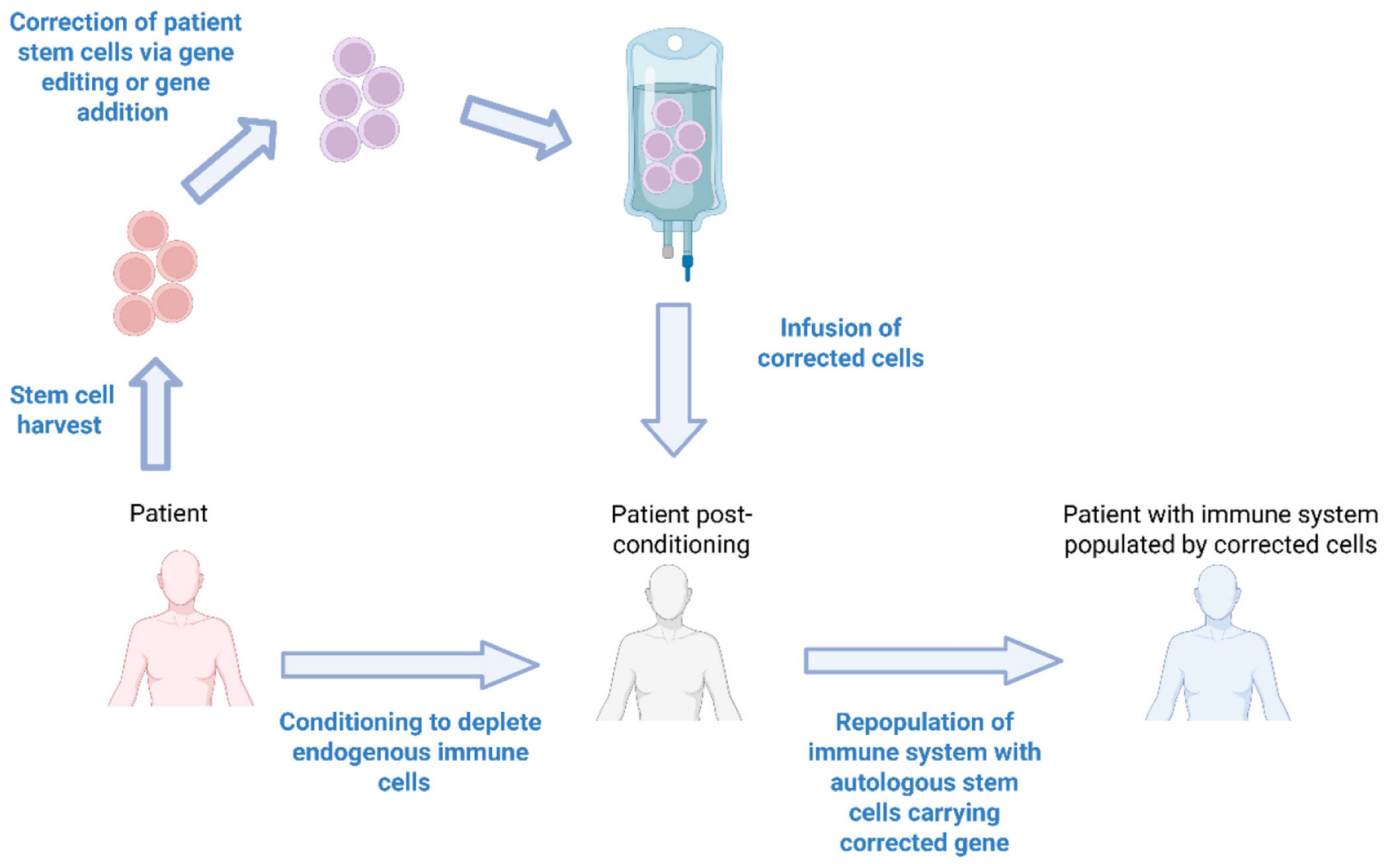
Autologous Haematopoietic Stem Cell Gene Therapies. Autologous HSC-GT involves harvesting the stem cells directly from the patient, which are then modified by gene addition or gene editing. Modified patient stem cells are then re-infused back into the patient, who in the meantime has undergone a conditioning regimen to deplete their endogenous immune cells. Following engraftment of the modified stem cells into the bone marrow of the patient, the newly introduced cells can repopulate the immune system with non-diseased cells. Created in BioRender. Torrance R (2025) https://BioRender.com/dmazkdh. HSC-GT: Haematopoietic stem cell gene therapies.

Autologous haematopoietic stem cell (HSC) gene therapies (GT) (HSC-GT) completely overcome the issues of donor availability and immunological complications (GVHD, immunosuppression-related infections, graft failure) associated with alloHSCT. HSC-GT involves harvesting the haematopoietic stem and progenitor cells from patients, typically via the apheresis of mobilized stem cells from the peripheral blood. Stem cells are then modified using gene addition/editing and returned to the patient following conditioning chemotherapy [Figure 1]. Modified patient HSCs home to the bone marrow and can repopulate all cell lineages with corrected progeny. HSC-GT does not require a donor and there is no risk of GVHD. Given the autologous nature, there is less risk of graft failure and a lack of requirement for immunosuppression following HSC infusion^[12]^. IEIs represent the ideal application for HSC-GT, given the clear link between genotype and disease phenotype and the defect being limited to cells that can be repopulated from corrected HSCs.

In this review, we discuss the development of HSC-GT for IEIs, from gene addition to gene editing and give a perspective on advances and future directions.

## Gene addition for inborn errors of immunity

**Figure 2:**
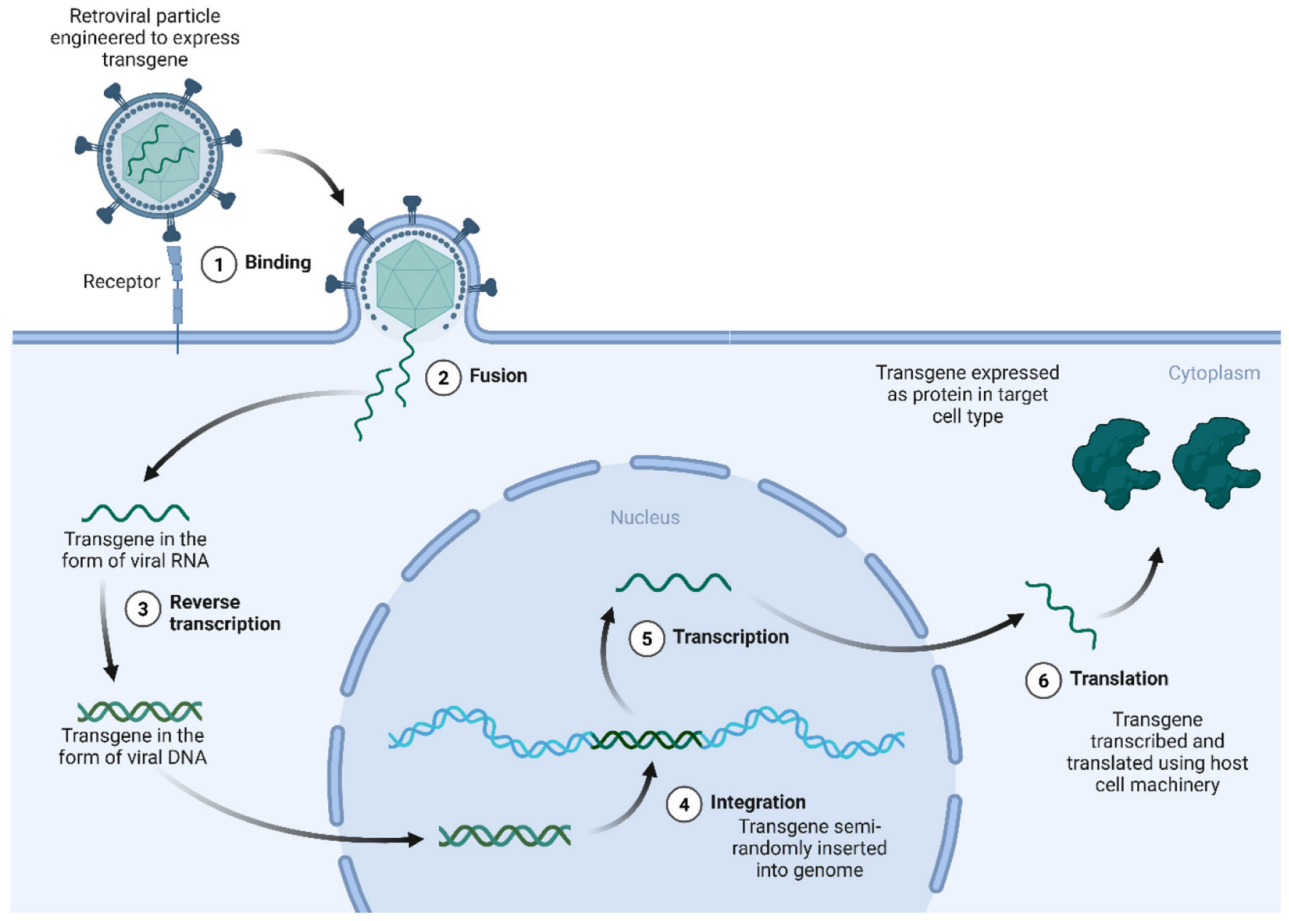
Gene addition for gene therapies using retroviral vectors. Gene transduction using retroviruses such as HIV-derived lentivirus or γ-retroviruses begins when virus particles enter the cell either by endocytosis or following direct fusion of the viral envelope protein with its cognate receptor on the membrane (1,2). Upon entry, the retroviral RNA genome is released into the cytoplasm where it is reverse transcribed by a viral reverse transcriptase (3) to produce cDNA which integrates semi-randomly into the host genome using a virally expressed integrase (4). While lentiviruses can transduce dividing and non-dividing cells, simple γ-retroviruses require the disappearance of the nuclear membrane during cell division, meaning that they are only able to successfully infect dividing cell types. Integrated sequences are then expressed using the host transcriptional (5) and translational (6) machinery. Promoter sequences either in the long-terminal repeat region of the virus, or more commonly for gene therapy approaches, from a mammalian promoter sequence incorporated into the genomic cargo, are used to regulate gene expression. Created in BioRender. Torrance R (2025) https://BioRender.com/thj9v37. cDNA: Complementary DNA; HIV: human immunodeficiency virus.

The concept of genetic modification of human cells as a therapeutic option originated in the early 1970s, when it was hypothesized that the introduction of exogenous DNA into cells bearing genetic defects could be a viable therapeutic strategy for patients with genetic disease^[13]^. At the time, attempts at exogenous gene transfer in human cells showed limited efficiency. The use of more efficient transformation methods, such as engineered retroviruses, revolutionized the field^[14–18]^. Genetic disorders caused by a loss of function in a given protein could now be corrected using viral vectors to introduce a new, functional copy of the gene into patient cells [Figure 2].

By 1990, γ-retroviral vectors had been successfully engineered to deliver complementary DNA (cDNA) encoding the adenosine deaminase gene (*ADA*) into the lymphocytes and bone marrow of patients with ADA-SCID. This provided the first proof-of-concept that GT could be a safe and effective therapeutic strategy for IEIs^[14,15]^. Following this success, over the next twenty years, a number of clinical trials examining safety and efficacy of GT using γ-retroviral vectors for IEIs were initiated, with promising results in ADA-SCID^[16,17]^, X-linked SCID (X-SCID)^[18–21]^, Wiskott-Aldrich Syndrome (WAS)^[22,23]^ and X-linked Chronic Granulomatous Disease (X-CGD)^[24,25]^.

However, despite improvement of clinical phenotypes in many cases, the use of γ-retroviral vectors led to insertional oncogenesis in a number of patients. For example, despite sustained clinical benefit in 17/20 patients initially treated in a GT trial for X-SCID, 6/20 patients developed T cell leukemia linked to preferential vector integration in close proximity to protooncogenes such as the LMI domain only protein 2 (LMO2)^[20,21,26,27]^. Similar serious adverse events were also seen in the WAS trial where 7/10 enrolled patients developed leukemia^[23]^, as well as in the Chronic Granulomatous Disease (CGD) trial where vector integration close to EVI1 was linked to the development of myelodysplasia with monosomy 7, a precursor to Acute Myeloid Leukaemia (AML)^[25]^.

Interestingly, the rates of oncogenesis in ADA-SCID patients following γ-retroviral vector GT have been much lower than in other primary immunodeficiency disorders (PIDs), with only one case of vector-related leukemia in over 75 patients treated to date^[28]^. The exact reasons for this remain unclear, but it is thought to be related to intrinsic ADA deficiency in epithelial cells restricting thymopoiesis, which may in turn reduce the risk of leukemogenesis through a decreased rate of precursor T-cell expansion^[29]^.

Due to the development of oncogenesis with γ-retroviral therapies, newer and safer self-inactivating (SIN) γ-retroviral vectors were developed, where the enhancer sequences within the long terminal repeat (LTR) were replaced with endogenous or lineage-specific promoters^[30]^. These vectors show equivalent efficiencies compared to γ-retroviral vectors, with no reports of insertional mutagenesis at follow-ups beyond 10 years in SCID-X1 and ADA-SCID^[26,29]^. However, γ-retroviral vectors have largely been replaced by lentiviral vectors, which offer safer & more efficient transgene delivery for IEI treatment. The improved safety profile of lentiviral vectors is due to their preferential integration in actively transcribed genes, rather than promoter or enhancer regions, reducing the risk of insertional mutagenesis. Indeed, to date, there have been over 30 early phase clinical trials utilising lentivirus-mediated gene addition both *in vivo* and *ex vivo* for IEIs, with a sustained clinical benefit (partial or complete) demonstrated in clinical trials for X-SCID^[31]^, WAS^[32,33]^, Leukocyte Adhesion Deficiency (LAD)^[34]^, X-CGD^[35]^ and ADA-SCID^[36]^ [Table 1]. Importantly, there have been no oncogenic transformations in any of these patients to date.

However, lentiviral gene addition is not suitable to correct all IEIs as it can only be used when the genetic mutation results in a missing or absent protein or where overexpression is tolerated or advantageous (e.g., ADA-SCID). Furthermore, the semi-random nature of transgene integration that results from lentivirus transduction does not typically result in physiological expression in all cells. This is particularly important for IEIs where tightly regulated gene expression is required, such as for CTLA-4 insufficiency^[59]^ or X-linked hyper IgM syndrome, where constitutive expression of the implicated gene CD40 ligand (*CD40L*) has been linked to lymphoproliferative disorders^[60,61]^. Furthermore, simple gene addition is not suitable for IEIs caused by dominant mutations, such as gain-of-function or dominant negative disorders, which make up approximately 27% of all IEIs^[1]^. For these indications, novel gene editing technologies may enable correction of the associated genetic defects while preserving the endogenous control machinery and gene expression profile^[26,62,63]^.

## Gene editing for inborn errors of immunity

To date, several gene editing technologies have been used for the precise alteration of DNA sequences in IEIs. These include nuclease-based gene editors, base editors (BEs), and prime editors (PEs). The class of editor most suitable for a given indication depends on several factors, including the type of desired edit, the properties of the targeted DNA sequence, and the disease context.

### Clustered regularly interspaced short palindromic repeats/Cas endonucleases

Three main classes of nucleases have been described for targeted genome engineering, each of which uses a targeting molecule (either protein or RNA) to guide a DNA nuclease to a specific region of the genome, where a double-stranded (ds) DNA break is created^[64,65]^. Historically, zinc-finger nucleases (ZFNs) and transcription activator-like effector nucleases (TALENs) which use protein moieties for targeting were utilized and demonstrated proof-of-principle of gene editing in human cells^[65–67]^. However, in recent years, the field has largely shifted towards using the Clustered Regularly Interspaced Short Palindromic Repeats associated with the Cas endonuclease (CRISPR-Cas) system for gene editing due to its ease of programmability^[68]^. CRISPR-Cas gene editing can be easily programmed by altering the guide RNA (gRNA) sequence, allowing targeting of the Cas endonuclease to different genomic loci based on complementary RNA:DNA base pairing. Indeed, following the first use of the CRISPR-Cas system to induce targeted double-stranded DNA (dsDNA) breaks *in vitro* in bacteria in 2011^[68]^, there has been an exponential rise in applications of CRISPR-Cas technology from bacteria to human cells^[69]^.

**Figure 3:**
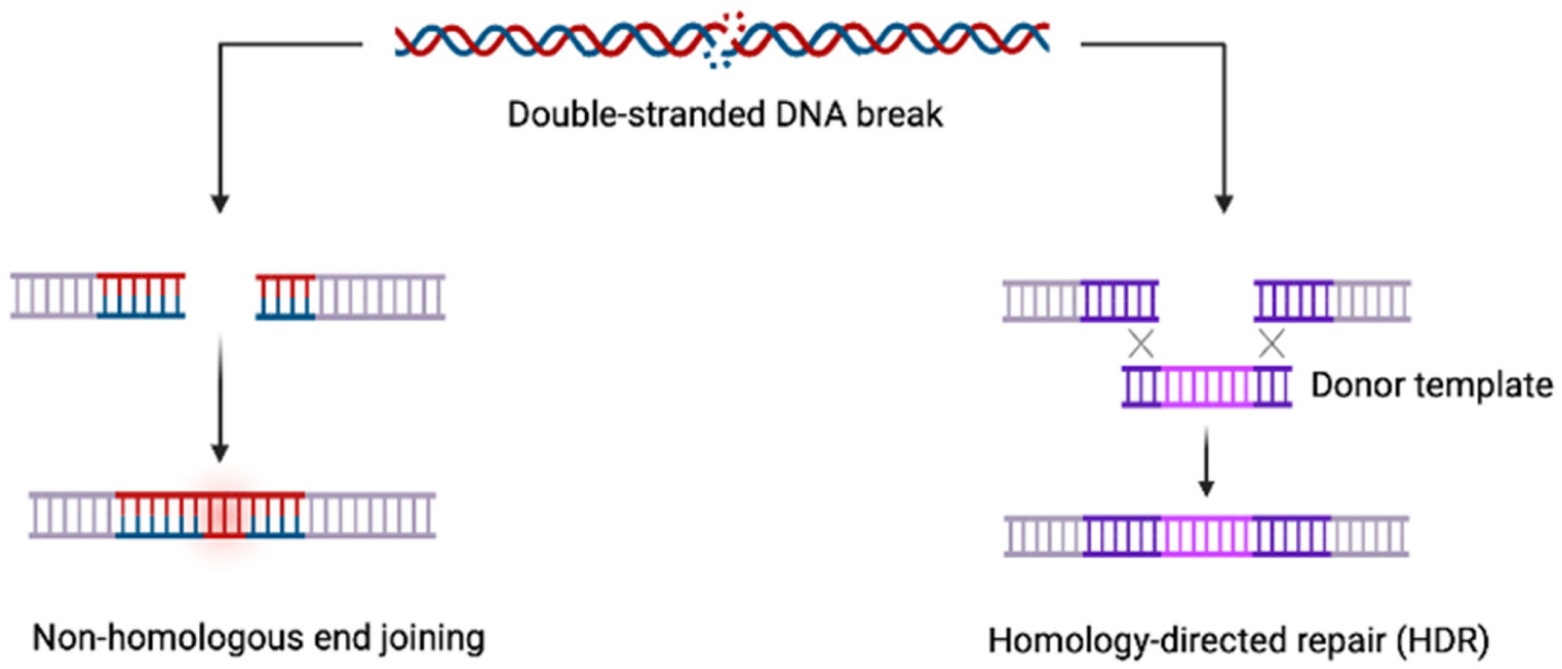
Mechanisms of repair of a dsDNA break. Following generation of a DSB by a CRISPR/Cas nuclease, the lesion is typically either repaired by either non-homologous end joining, which is highly error prone and often leads to gene disruption, or by homology-directed repair which can lead to precise edits at the cut site. Created in BioRender. Orf K (2025) https://BioRender.com/kfj1a7h. CRISPR: Clustered regularly interspaced short palindromic repeats; dsDNA: double-stranded DNA; DSBs: double-strand DNA breaks.

Regardless of the nuclease used, following the formation of a dsDNA break, the primary repair mechanism in human cells is non-homologous end joining (NHEJ) [Figure 3]^[70]^. This results in semi-random insertions and deletions (indels) of bases at the site of the dsDNA break, often leading to frameshifts, an alteration in the coding sequence and thus gene knockout^[71]^. However, in the presence of a suitable “donor” template with homology to the DNA either side of the cut site, homology-directed repair (HDR) can take place, leading to the insertion of new sequences at the location of the break^[72–74]^ [Figure 3]. Whereas NHEJ can occur at any time during the cell cycle, HDR only occurs during the S or G2 phases of the cell cycle, and thus typically happens at a lower frequency^[75,76]^. This is in contrast to base and prime editing (discussed below), which utilize repair mechanisms that are not cell cycle-dependent, allowing efficient gene editing of cycling and non-cycling cells.

Several different types of repair templates can be utilized for HDR-based gene editing. For example, Adeno-associated virus type 6 (AAV6) and integrase-deficient lentiviruses (IDLVs) have been used to allow the targeted integration of large templates^[77–80]^. At present, there is no clear indication for preferential use of AAV6 or IDLVs in specific IEIs; both have shown utility in preclinical settings, and choice typically reflects evolving safety data, vector characteristics, and the manufacturing capabilities of individual institutions. Recent reports have also demonstrated successful editing using non-viral donor templates. For example, single-stranded oligodeoxynucleotides (ssODN) can generate therapeutic levels of HDR^[81]^. Ultimately, if HDR is appropriate for the therapeutic application, which repair template to use depends on a number of different factors including the type of edit to be installed (small edit *vs*. large insertion), the target cell type (and the degree of toxicity that the cell can tolerate), the cost and complexity of manufacture, and the off-target risks.

### Base editing

**Figure 4:**
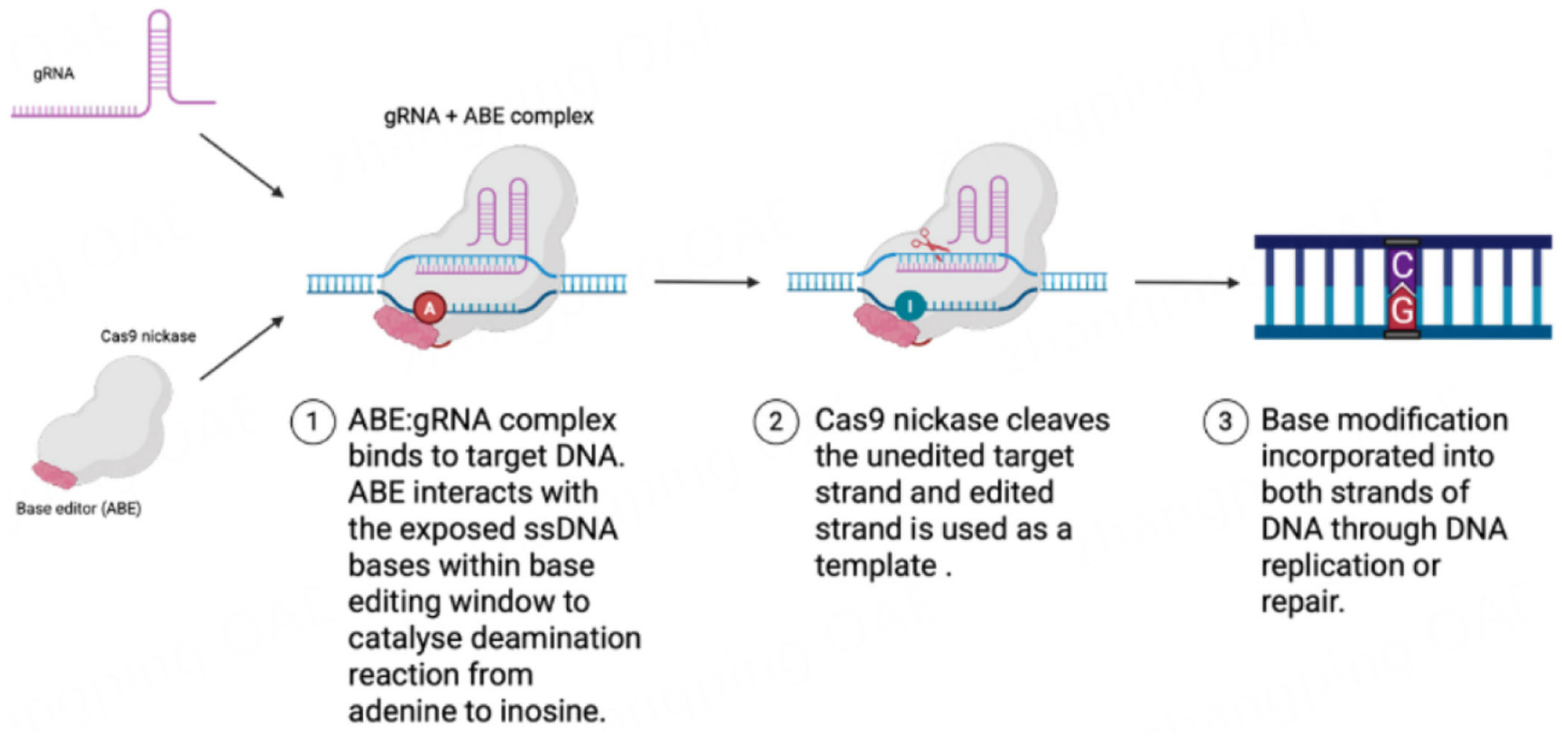
Schematic diagram showing the mechanism of adenine base editing. A gRNA targets a Cas nickase tethered to an adenine/cytosine deaminase to a specific sequence within the genome. There, specific deamination of target bases within a base editing window takes place, resulting in A:T - > G:C or C:G - > T:A transition mutations. Created in BioRender. Orf K (2025) https://BioRender.com/udxwt5j. ABEs: Adenine base editors; gRNA: guide RNA.

Base editing is a further development of the CRISPR/Cas technology that allows all four possible transition point mutations (A- > G, T- > C, C- > T, G- > A) to be performed, without the creation of double-strand DNA breaks (DSBs). BEs consist of a Cas nickase (nCas) fused to an adenine/cytosine deaminase domain^[82]^. As in the CRISPR-Cas system described above, the gRNA directs the nCas to the target sequence by binding to the target DNA strand, and producing a single-stranded DNA (ssDNA) substrate for the deaminase which deaminates specific nucleotides within this sequence [Figure 4]^[82]^. The precise editing window varies depending on the BE but typically spans positions 4 to 8 of the protospacer sequence for BEs based on SpCas9, where the protospacer adjacent motif (PAM) is located at position 21 onwards^[82]^. After conversion of target bases in the editing window, the nCas creates a single-stranded break on the non-deaminated strand which stimulates repair using the base-edited ssDNA strand as a template.

One of the major requirements for efficient base editing is the availability of a suitable PAM sequence that places the target base in an appropriate base editing window. Early BEs were exclusively based on SpCas9 (PAM=NGG) which theoretically permits the correction of just 26% and 28% of annotated, pathogenic transition mutations in ClinVar with cytosine base editors (CBEs) and adenine base editors (ABEs), respectively^[82]^. However, with the evolution of SpCas9 variants to accommodate different PAM sequences, the use of deaminase domains with alternative sequence specificities and editing window widths, and the use of alternative Cas enzymes (e.g., SaCas9, LbCas12a), around 95% of all pathogenic transition mutations in ClinVar are now targetable by base editing^[82–87]^ [Table 2]. Selection of a particular BE/nCas combination depends on several factors, including the required base conversion efficacy, PAM availabilities and the presence of possibly deleterious bystander edits. Relative editing efficiencies and potential advantages/disadvantages of the different editors are largely dependent on the target sequence and indication.

### Prime editing

**Figure 5:**
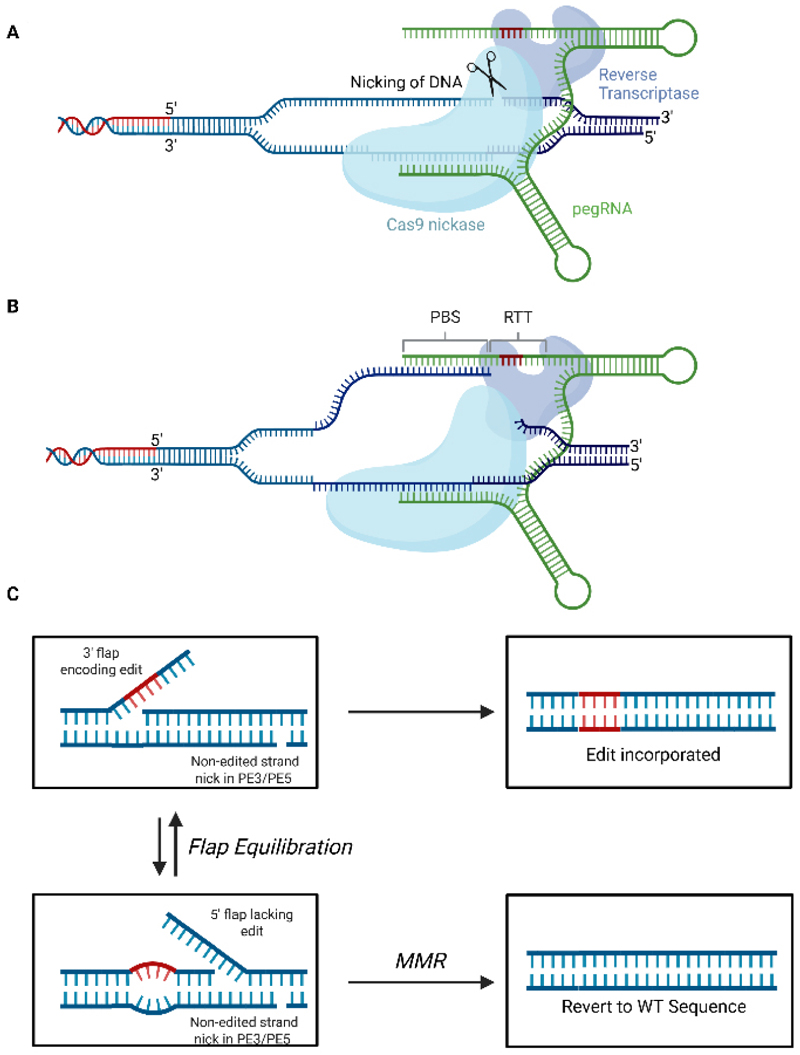
Mechanism of Prime Editing. (A) Prime editing initiates when the protospacer of the pegRNA binds to its cognate site within the genome before a nick in the phosphodiester backbone is created on the target strand; (B) Following nicking, the PBS anneals to the DNA flap with the resulting double-stranded DNA duplex acting to prime reverse transcription using the RTT as a template; (C) This produces a 3’ flap containing the desired edit which then undergoes flap equilibration with the 5’ flap lacking the edit. Degradation of the 5’ flap and ligation of the 3’ flap into the genome will result in the incorporation of the edit. Recognition of the mismatch between the 3’ flap containing the edit and the unedited by the MMR DNA repair machinery can prevent incorporation of the edit. A nick to the non-edited strand in the PE3 and PE5 systems and/or expression of a dominant negative mismatch repair protein MLH1 in PE4 and PE5 systems can bias DNA repair in favour of incorporation of the edit. RTT: Reverse transcription template; pegRNA: prime editing guide RNA; PBS: primer binding site; MMR: mismatch repair; WT: wildtype. Adapted from Doman *et al*.^[92]^, Created in BioRender. Torrance R (2025) https://BioRender.com/06c7els.

Although BEs can correct most pathogenic single nucleotide polymorphisms (SNPs), they cannot correct all types of mutation (e.g., substitutions, indels)^[91]^. PEs are able to mediate all single-nucleotide conversions, as well as small insertions and deletions without creating DSBs^[91]^. PEs consist of reverse transcriptase (RT) enzyme fused to a nCas domain [Figure 5]. A prime editing gRNA (pegRNA) can both direct the PE to the genomic target locus and encode the desired edit. As with nuclease-mediated gene editing and base editing, prime editing initiates with base pairing of the protospacer sequence to the target site in the genome and subsequent nicking of the non-protospacer bound strand^[92]^. Following nicking, the primer binding site within the pegRNA anneals to the nicked strand forming an RNA/DNA duplex that can be used to prime reverse transcription, with the reverse transcription template (RTT; also within the pegRNA) serving as a template. As reverse transcription occurs, the flap is extended, and the edit is incorporated. The edited 3’ flap then competes with the unedited 5’ flap to anneal to the unedited complementary strand. Newer iterations of PEs (e.g., PE3/PE5) utilize an additional single-guide RNA (sgRNA) to nick the complementary strand of DNA, stimulating degradation of the unedited strand via the endogenous mismatch repair (MMR) machinery^[91,93]^.

## Current clinical and preclinical applications of gene editing for IEI

Several approaches utilizing the genome editing technologies described above have been developed for the treatment of different IEIs. The vast majority of these applications have been for those disorders caused by homozygous or heterozygous loss-of-function (LOF) mutations where gene editing offers a rival approach to gene addition, with the additional benefit of allowing tightly regulated, physiological expression due to the preservation of the endogenous control machinery^[59–61]^. These approaches for IEIs caused by LOF mutations typically involve the use of CRISPR/Cas9 nucleases to induce a dsDNA break within the endogenous gene, before a therapeutic (full or partial) transgene is introduced at the cut site by HDR. Indeed, such an approach has been successfully applied in multiple IEIs including SCID-X1, X-linked hyper IgM, recombinase activating gene 1 (RAG1) SCID, recombinase activating gene 2 (RAG2) SCID, WAS, X-linked agammaglobulinemia, Interleukin-7 receptor α (IL-7Rα) SCID, immunodysregulation, polyendocrinopathy, enteropathy, X-linked (IPEX) Syndrome, X-CGD, X-linked immunodeficiency with Magnesium defect, Epstein–Barr virus infection, and Neoplasia (XMEN) Syndrome, X-linked lymphoproliferative disease and CTLA-4 insufficiency [Table 3].

However, in some cases, a knock-in approach, such as the one described above, is not suitable or feasible, and a mutation-specific approach is more appropriate. This may be true, for example, when high levels of editing are required to correct the disease phenotype. Given that CRISPR/Cas9 HDR is cell cycle-dependent, HDR efficiencies in long-term HSCs are typically limited to less than 50%, and frequently < 30% depending on several factors including the target site, the size of the repair template and the viability of edited cells. Additionally, some therapeutic transgenes may be too large to use common HDR delivery templates such as AAV6, necessitating alternative means of delivery.

In recent years, several mutation-specific strategies have been developed for IEIs. For example, Dettmer-Monaco *et al*. utilized a dual guide RNA strategy to specifically excise a mutant sequence in intron 26 of the *Unc13d* gene that otherwise encoded for a cryptic splice site in order to restore a functional, polyclonal T cell response in a murine model of haemophagocytic lymphohistiocytosis^[108]^. Similarly, De Ravin *et al*. have shown that repair of the most frequent X-CGD-causing mutation c.676C > T in the Cytochrome b-245, beta chain (*CYBB*) gene using CRISPR/Cas9 and an ssODN resulted in sustained production of mature human myeloid and lymphoid cells following transplantation of edited X-CGD human cells bearing this mutation into nonobese diabetic (NOD) SCID mice^[81]^.

Increasingly, newer gene editing technologies such as base and prime editing are also being applied to permit mutation specific correction without the production of DSBs, thus preventing mixtures of uncontrollable indel byproducts from forming, reducing p53 activation and reducing the risk of chromosomal translocations and other gross genomic aberrations^[110]^. Indeed, recently, base editing of the same prototypical c.676C > T X-CGD mutation described above was successfully achieved at efficiencies 3.5-fold greater than the CRISPR/Cas + ssODN approach that had been previously trialled, with no large chromosomal rearrangements detected and an improved reported genotoxicity profile^[106]^. Similarly, base editing has recently been used to correct the founder c.202C > T mutation in the *CD3D* gene, which is causative of CD3δ SCID, leading to the production of mature, functional T cells in artificial thymic organoids (ATO)^[105]^. Finally, Prime Medicine has recently announced its work demonstrating that prime editing is able to successfully correct defects in long-term hematopoietic stem cells (LT-HSCs) derived from p47phox-deficient CGD patients^[107]^.

## T cell gene therapy for IEI

For most IEIs, correction of the HSC compartment is needed for multilineage expression and robust immune reconstitution. HSCs have the advantage of their inherent property of self-renewal, thus potentially providing a source of corrected immune cells for the lifetime of the recipient^[36,79,111]^. However, in several IEIs, the defect is limited to the lymphoid compartment. In diseases such as IPEX syndrome, CD40 ligand deficiency, CTLA-4 insufficiency and X-linked lymphoproliferative syndrome (XLP), it is defects in T cells (or T cell subsets) that result in the disease phenotype^[59,80,94,112,113]^. In diseases such as these, correction of the T cell compartment alone may improve the disease phenotype whilst offering advantages over HSC GT^[59]^.

There are several advantages of a T cell GT approach over autologous CD34+ cells as the starting material. Firstly, the conditioning needed to engraft engineered CD34+ cells is more toxic than the lymphodepletion required prior to a T cell infusion^[114,115]^. We also know from studies exploring the role of alloHSCT in some T cell mediated IEIs such as CTLA-4 insufficiency that there is a high risk of stem cell engraftment failure due to the inflammatory environment present in patients with this disease^[116]^.

Secondly, as T cells are terminally differentiated cells, the genotoxic risks from gene editing procedures may be less than in CD34+ cells^[94,117,118]^. CRISPR/Cas9 gene editing is a new technology, and whilst Advanced Therapy Investigational Medicinal Products (ATIMPs) using this are in clinical trials or approved for use in humans [e.g., Casgevy (exagamglogene autotemcel)], there are potential risks of genotoxic events^[114]^. There have been no concerns regarding CRISPR/Cas9 mediated homology directed repair in T cells and several trials of T cells engineered using this technology are currently recruiting (e.g., NCT05643742).

Whilst the long-term persistence of autologous T cell GT for IEIs is not yet known, extrapolating from experience with autologous chimeric antigen receptor (CAR)-T therapies and early T cell GT studies (in ADA-SCID), it is likely to be long-term^[119]^. Experience from T cell cellular therapy suggests that genetically modified T cells can persist for > 10 years if enough T stem cell memory cells are included in the therapeutic product^[119]^.

Robust preclinical proof-of-principle of T cell GT approaches (both viral gene addition and gene editing) have been published for IPEX syndrome, CD40 ligand deficiency, XLP and CTLA-4 insufficiency^[52,59,80,94,120,121]^. Proof of principle in humans is awaited but a trial of the lentiviral CD4+ T cells approach for IPEX syndrome is being assessed in a phase I clinical trial (NCT05241444) and a trial of a lentiviral T cell GT approach for XLP is expected to open in the UK later in 2025^[52]^. Whilst it remains to be seen whether T cell correction can offer lasting improvement in disease phenotype in humans, in select IEIs, preclinical data suggests that this is a promising and potentially less toxic approach than HSC correction. While T cell-based GTs hold considerable promise, long-term risks remain incompletely defined. In particular, uncertainties exist regarding persistence and the potential for functional exhaustion, which can only be fully assessed in human clinical trials. Several such studies are expected to open soon and will be critical in addressing these unknowns.

## Gene therapy for adolescents and adults with IEI

As data for efficacy of alloHSCT has accumulated in the non-SCID IEI setting, successful GT approaches for non-SCID IEIs have been published^[35,44,122,123]^. In recent years, increasing numbers of adolescent and adult patients with non-SCID IEIs have been treated with alloHSCT, raising the question of whether GT could be applied to older patients^[124]^. As they have lived with their IEIs for several years, these individuals often have more comorbidities, making them higher-risk transplant candidates^[10,125]^. Patients with uncontrolled autoinflammation prior to transplant have an increased risk of graft failure and GVHD^[10]^. Older patients may therefore stand to benefit most from less toxic, autologous GT approaches.

Due to thymic involution, there were concerns that autologous HSC-GT may not result in robust multilineage immune reconstitution in older patients with IEIs. In 2017, a 30-year-old patient with Wiskott-Aldrich syndrome, who had severe manifestations of the disease but lacked a suitable donor for alloHSCT, was successfully treated with autologous HSC GT^[126]^. Following reduced intensity conditioning and infusion of autologous lentiviral-modified HSCs, robust multilineage engraftment of transgene positive cells was observed, and the patient was able to cease immunoglobulin replacement^[126]^. Since this first demonstration that autologous GT was a viable strategy in adults with IEIs, subsequent clinical trials have recruited adult patients. In the trial of lentiviral transduced HSCs for X-CGD, 6 of 9 patients were adults^[35]^. Upcoming trials of novel GT approaches for IEIs will recruit both older patients and children (e.g., NCT06876363).

## *In vivo* gene therapy

**Figure 6:**
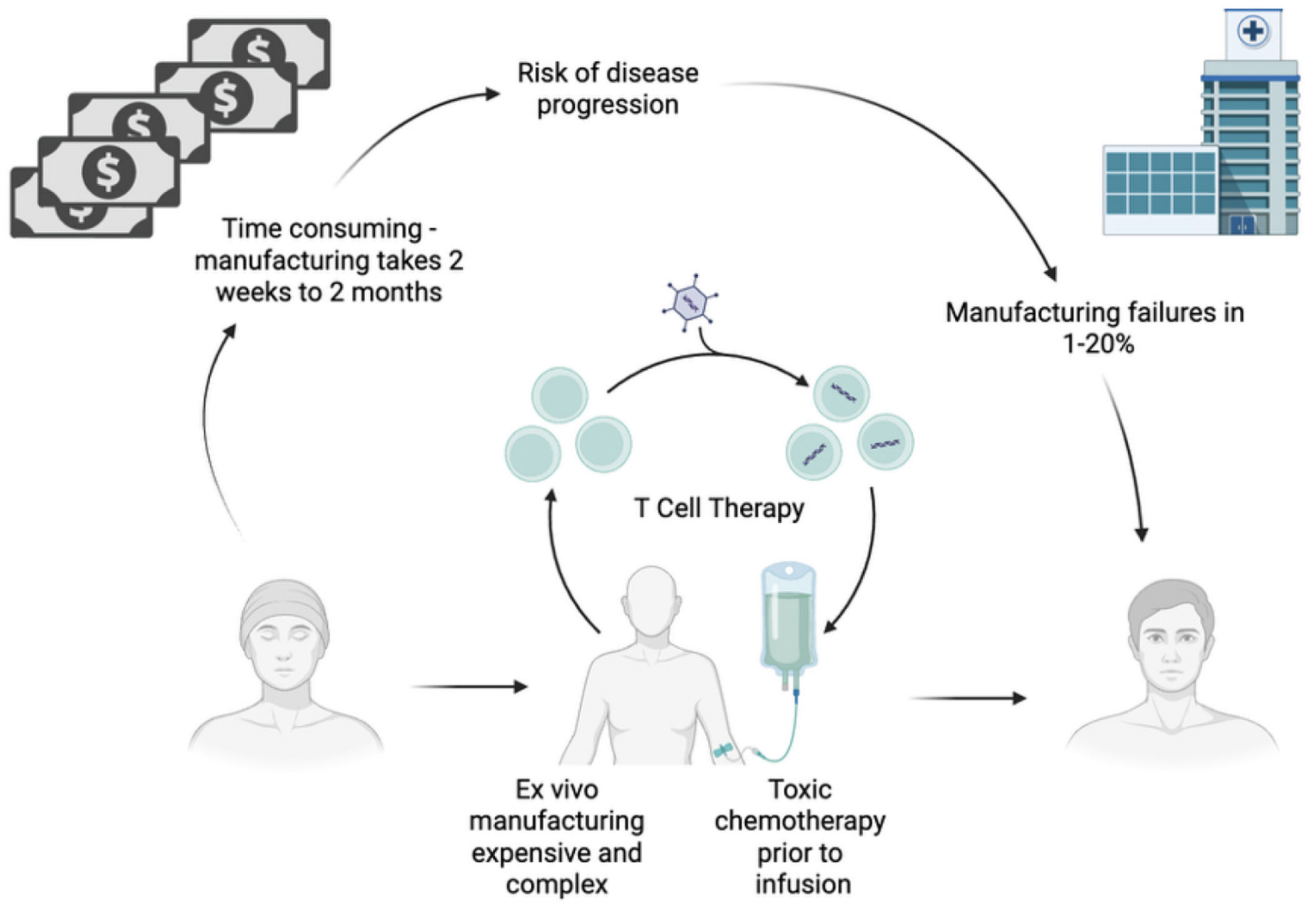
Potential limitations of *ex vivo* T cell therapy relative to *in-vivo* T cell therapy. Created in BioRender. Fox TA (2025) https://BioRender.com/kkpbc4l.

Despite remarkable clinical results, *ex vivo* GT has major limitations. The need for a bespoke cellular product, harvesting of HSCs from the patient and *ex vivo* manufacturing all make GT expensive and complex to deliver [Figure 6]. *In vivo* GT has the potential to transform GT for IEIs from a unique cellular product to an off-the-shelf drug. This would dramatically reduce costs, complexity of delivery and toxicity associated with GT for IEIs.

*In vivo* GT requires a delivery platform that is non-toxic, non-immunogenic and can target a specific tissue. The main delivery technologies can be divided into virus or virus-like vectors (also known as enveloped delivery vehicles) and lipid nanoparticles (LNPs). Both technologies are in use in human clinical trials of *in vivo* GTs for other diseases. Two notable examples, at the advanced stage of clinical development, include adeno-associated virus-based therapies for haemophilia^[127,128]^ and lipid-nanoparticle CRISPR therapy for congenital amyloidosis^[129]^. Whilst these examples demonstrate that organ-specific transgene transduction and gene editing can be safely and effectively performed *in vivo*, both approaches target the liver. The liver contains 10%-15% of the body’s circulating blood volume making it an attractive and accessible target for *in vivo* gene delivery vectors administered intravenously^[130]^.

Targeting HSCs *in vivo* is more challenging due to both the physical location of the cells and the immune microenvironment of the bone marrow niche. Compared to the liver, HSCs are a rare population of cells (50,000-200,000 cells are estimated to be actively involved in haematopoiesis)^[131]^. Whilst both viral and non-viral delivery approaches targeting HSCs *in vivo* are being pursued, viral delivery platforms are at a more advanced stage of development. Researchers at the University of Washington have developed an HSC-tropic, helper- adenovirus-based gene transfer vector system. They have demonstrated that this platform can be used to perform base editing (CBE and ABE) and transgene integration (Sleeping Beauty transposase system) of HSCs *in vivo* in mice^[132]^. Prior to IV administration of the viral vectors, HSCs were mobilised into the peripheral blood using G-CSF/AMD3100^[132,133]^. In these studies, BEs were delivered *in vivo* to downregulate *BCL11A* for the treatment of haemoglobinopathies, but the same technology could also be applied to treat IEIs^[132,133]^.

One of the challenges of targeting HSCs in the peripheral blood is that vectors can be sequestered if the protein on the cell of interest is shared by other cell types. This phenomenon was observed in CD46-targeted adenovirus vectors in non-human primates as CD46 is also expressed on erythrocytes in this setting^[134]^. Another is that only a fraction of the mobilised CD34 cells home back to the bone marrow following transduction^[134]^. These challenges have been addressed in several ways including through targeting of a receptor that is only expressed on HSCs (e.g. desmoglein 2) and by using the vector to transiently overexpress CXCR4 which improved repopulation and migration capacity of the HSCs. Such innovations resulted in transduction of over 5% of HSCs in non-human primates^[135]^.

Several groups are working on HSC-targeted LNPs and the last few years have seen some exciting progress in this field^[136,137]^. CD117-targeted LNPs have been demonstrated to deliver mRNA *in vivo* in mice^[136]^. Interestingly, the authors of this study delivered mRNA coding for pro-apoptotic PUMA (p53 up-regulated modulator of apoptosis) to deplete HSCs with the proposed clinical application of non-genotoxic conditioning^[136]^. Such technology (if the results can be replicated in non-human primates and humans) could have several applications to the IEI field.

*In vivo* HSC GT is set to first be attempted in humans to treat IEIs. Ensoma Therapeutics has developed a virus-like particle (VLP) platform based on the helper-dependent adenovirus 3/35++ technology previously described and is using it to deliver the *CYBB* gene to HSCs for the treatment of X-CGD^[138]^. Ensoma’s approach (a VLP product called EN-374) consists of two VLPs: one containing a Sleeping Beauty 100X transposase and Flp recombinase and another with a transposase, myeloid promoter and a copy of the *CYBB* gene. The second VLP also contains a methylguanine methyltransferase (MGMT) marker containing the P140K mutation which enables engineered HSCs to be enriched by the administration of temozolomide^[138]^. Ensoma Therapeutics is expected to open a trial of EN-374 in the United States and Europe in 2025 (NCT06876363). Patients will undergo mobilization of HSCs, followed by IV injection of the VLP product EN-374 with simultaneous immune prophylaxis. Patients will receive temozolomide following VLP infusion to enrich the engineered HSCs *in vivo*. The results of such a trial will be eagerly awaited, as if it is successful, similar approaches could be used for many other IEIs in which correction of the HSC compartment is desired.

*In vivo* GT represents the next frontier in the GT field. Given its transformational potential to reduce toxicity, costs, and complexity, it could ultimately reach many more patients than *ex vivo* approaches. By removing the need for bespoke cell harvesting, *ex vivo* manufacturing, cryostorage, and transplantation expertise, *in vivo* GT offers the potential to broaden access to patients in low- and middle-income countries. However, whether this promise can be realized will depend on parallel progress in addressing regulatory, infrastructural, and cost barriers that continue to limit equitable deployment of advanced therapies. Indeed, progressing *in vivo* GT worldwide is contingent on navigating stringent regulatory hurdles, including requirements for detailed biodistribution, long-term safety, and immunogenicity data. Platforms such as Ensoma’s VLP system are addressing these challenges through stepwise trial designs and close regulatory engagement, with forthcoming clinical studies expected to provide critical insights.

## Status of gene therapy for IEI

Despite the promising preclinical development and clinical trials of GT approaches outlined in this review, these therapies remain experimental, and access to them is almost exclusively through clinical trials. In the United States, no GTs for IEIs are approved by the Federal Drug Administration (FDA). In the European Union a single GT, Strimvelis, a γ-retroviral-based HSC therapy for ADA-SCID is approved by the European Medicines Agency (EMA)^[139]^. In 2024, a patient treated with Strimvelis developed T cell leukemia 4.7 years after treatment because of retroviral integration at the LMO2 locus followed by acquisition of a complex set of somatic mutations^[28]^. The risk of mutagenesis is well characterized with γ-retroviral vectors but in ADA-SCID, it is mitigated in part by disease-related factors. After extensive investigation the EMA concluded that the risk/benefit analysis for Strimvelis remains favourable, and it remains approved in Europe albeit with robust monitoring arrangements^[28]^. The evidence for autologous GT is strongest for ADA-SCID with 100% overall survival and 90%-95% engraftment^[111,140]^. Recently updated treatment guidelines for the management of ADA-SCID recommend consideration of autologous GT for the condition when a matched family donor is not available^[140]^. This makes ADA-SCID, the only IEI for which GT is considered a standard of care. However, aside from Strimvelis which is a fresh cell product necessitating travel to the only treatment centre in Milan, GT can only be accessed through clinical trials. As of October 2025, there were trials of lentiviral HSC GT for ADA-SCID open and recruiting IN the United States (NCT05432310) and China (NCT03645460).

In other SCID IEIs, access is also limited to clinical trials. For X-SCID, there are trials of lentiviral vector approaches open and recruiting in the UK (NCT03601286), USA (NCT01306019, NCT03311503) and China (NCT03217617). Several trials are open and recruiting As of October 2025 for Artemis SCID (NCT03538899, NCT05071222) and RAG1-deficient SCID (NCT04797260). In the non-SCID context, there are trials open of GT for X-linked (NCT06559176 - prime editing of HSCs) and autosomal recessive (NCT05207657) CGD. A trial of a lentiviral vector modified T cell GT approach for IPEX syndrome is open and recruiting in the USA (NCT05241444). Several trials of GT (HSC and T cell) in other IEIs are expected to open soon (e.g., NCT06876363, NCT06876363).

The lack of availability to GT despite strong efficacy and safety data has now been recognized as a major issue for the field. The reasons are complex and are discussed in the next section along with potential solutions.

## Advances in gene therapy for IEI

### Improving access to gene therapy

GT is not used in routine clinical practice. The reasons are twofold. Firstly, simultaneous advances in alloHSCT have reduced some of the impetus to develop and deliver autologous GTs. These advances, coupled with the current higher costs of GT compared to alloHSCT, have reduced the attractiveness of GT in financially constrained healthcare systems. The development of haploidentical transplant protocols has dramatically increased donor availability^[141,142]^. Reduced intensity conditioning protocols, coupled with targeted drug dosing, have reduced the toxicity associated with the procedure^[143]^. Advances in GVHD prophylaxis including graft manipulation have reduced GVHD and improved infection prophylaxis with reduced morbidity from infectious pathogens^[144–147]^.

Despite the laudable advances in alloHSCT, autologous GT offers clear advantages, including universal applicability (no need for a donor) and the absence of GVHD risk. It also has a better safety and efficacy profile, although the number of patients treated remains small. The risks of mortality and morbidity associated with alloHSCT remain unacceptably high, particularly in the absence of a sibling donor^[7,142,143,148]^. Take ADA-SCID as an example. Five-year overall survival for alloHSCT preceded by enzyme replacement therapy was 79.6% compared to 100% for autologous GT^[111]^. Headline overall survival rates do not tell the whole story - reduced morbidity to both patient (and potentially an alloHSCT donor) due to lack of GVHD and reduced infectious complications due to a lack of immunosuppression all lead to improved quality of life in patients receiving GT versus alloHSCT^[36]^. As clinical experience with GT increases, the body of evidence to support the use of these more expensive autologous therapies will increase which will ultimately lower the costs for the health system.

However, even with increasing clinical data demonstrating improved survival and quality of life following GT compared to alloHSCT, a second reason explains the lack of widespread use of GT for IEIs. This reason is non-clinical and relates to the widespread commercial disinvestment in GTs for rare diseases that has occurred over the last decade^[149–151]^. In the rare and ultra-rare disease space, the costs of developing a novel GT product to the point of achieving market authorization are prohibitive and unattractive to for-profit pharmaceutical companies. There is now widespread recognition that in the rare disease space the current for-profit model of drug development is not fit for purpose.

Initiatives in both the United States and Europe are trying to address the challenge of access to GT for rare diseases. In Europe, the cross-border AGORA consortium (access to GT for rare diseases) was founded in 2022^[149]^. AGORA is working with key stakeholders from across the field to address current barriers to access. A similar taskforce has been established in the United States^[152]^. At present, international initiatives are largely region-specific, focused either in Europe or the United States due to differing regulatory frameworks; however, the issue of access is regularly discussed in forums such as the American Society of Gene and Cell Therapy (ASGCT) and European Society for Gene & Cell Therapy (ESGCT), which provide opportunities to share experience and align approaches across jurisdictions.

Some proposed solutions being explored include harmonised infrastructure for production, platform technology approvals and recognition of approvals across jurisdictions, standardized regulatory approach across different countries and data sharing to streamline safety assays^[150]^. Ultimately, it is anticipated that the use of non-traditional manufacturing and distribution models will be needed, for example, where an academic medical centre delivers a therapy on a per-patient basis under the European Union’s Hospital Exemption policy^[149,152]^. Many leading centers in the GT field, such as Great Ormond Street Hospital in London and San Raffaele in Milan, already operate Good Manufacturing Practices (GMP) manufacturing facilities, which could serve as prototypes for producing therapies at reduced cost within these emerging non-traditional models.

### Non-genotoxic conditioning and epitope editing

Two major advances in the cell and GT field are expected to further increase access to *ex vivo* GT for IEIs by reducing the toxicity, complexity and costs of treatment: the development of non-genotoxic conditioning and the use of epitope editing to enable selective enrichment of engineered cells^[153–155]^. There are two main targets being explored for targeted immunotherapeutic conditioning approaches, c-Kit (CD117) and CD45^[155–157]^. Both antigens are broadly expressed on HSCs (CD45 30 times more than c-kit^[155]^). Approaches for both targets have demonstrated promising preclinical efficacy^[157,158]^. IEIs are a desirable first target indication for these approaches as in the non-malignant setting the anti-neoplastic effect of genotoxic agents is unwarranted. Both CD45 and c-Kit targeted approaches have been assessed in clinical trials in the alloHSCT setting for IEI. A total of 16 IEI patients were treated with a CD45 targeted antibody (in combination with fludarabine, cyclophosphamide and alemtuzumab for immunosuppression); 15 of these engrafted (94%) and 11 (69%) achieved full or high level chimerism in the myeloid and lymphoid lineages, suggesting that the approach can facilitate engraftment and warrants further investigation^[159]^. Another trial of single-agent c-kit targeted antibody JSP191 is under investigation in SCID patients (NCT0293064)^[160]^. Whilst successful engraftment has been observed, several patients did not achieve robust multilineage engraftment and as such, it is not yet clear whether this approach will be successful^[160]^. Anti c-kit or anti-CD45 CAR T cells for conditioning are also being investigated with the hypothesis that they may be able to more potently clear HSCs compared to antibody or antibody drug conjugate (ADC) approaches^[161,162]^. Although preclinical results show promise, such approaches may carry additional toxicities and have yet to be assessed in a human clinical trial.

Whilst non-genotoxic conditioning has shown mixed results in clinical trials a parallel advance has the potential to improve the efficacy of non-genotoxic conditioning is epitope editing. Epitope editing refers to the use of base or prime editing to change several amino acids in the antibody-binding epitope of a protein. This creates “stealth receptors” which have preserved function but shield the cell from targeting with an antibody or CAR. Proof-of-principle of epitope editing of HSCs at several loci including CD45 and c-kit has now been demonstrated^[153,162]^. Non-genotoxic conditioning in combination with epitope editing may enable selection of an engineered population of cells facilitating engraftment. Such approaches are currently being investigated.

## Conclusions

Autologous GT has the potential to be an effective, safe, durable curative therapy for many IEIs, with less toxicity than alloHSCT. Both viral gene addition and gene editing can be used to correct or engineer autologous HSCs or T cells for therapeutic benefit. The choice of engineering technology and starting material depends on the target disease but we now have a versatile GT toolbox which can be applied to many IEIs. Autologous GT is now recommended as a standard of care for patients with ADA-SCID who lack a family donor. Despite this remarkable progress and recognition of clinical efficacy in treatment guidelines, access to GT for IEIs is almost exclusively restricted to clinical trials, in just a few treatment centres worldwide. Due to the rare nature of IEIs, the commercial development of GT approaches is not attractive to for-profit companies, which limits patient access to effective autologous therapies. Developments in GT, particularly *in vivo* delivery, may improve commercial interest, but issues surrounding marketing authorisation, costs, and regulatory hurdles need to be addressed before GT can emerge as a major therapeutic option. As the field advances, ensuring that the benefits of GT extend beyond early adopters and high-income settings is an ethical imperative, so that transformative treatments are accessible to all patients who need them.

## Figures and Tables

**Table 1:** Gene therapy clinical trials in progress for IEIs

Disease	Target gene	Vector	Clinical trials (Interventional)	Reference
γ-retroviral, SIN γ-retroviral, adenoviral and lentiviral				
ADA-SCID	*ADA*	γ-retroviral Lentiviral *(ex-vivo)* Lentiviral *(in-vivo)*	NCT00599781*; NCT00598481*; NCT00018018*; NCT01279720*; NCT00794508*	[16,37–41]
			NCT01380990*; NCT01852071*; NCT02022696*; NCT02999984*; NCT03765632*; NCT05432310	
			NCT03645460	[36]
X-linked SCID	*IL2RG*	γ-retroviral SIN γ-retroviral Lentiviral *(ex-vivo)* Lentiviral (*in-vivo*)	NCT00028236*	[42,43]
			NCT01410019*; NCT01175239; NCT01129544*	[29]
			NCT01512888; NCT01306019*; NCT03315078; NCT03311503; NCT03601286; NCT04286815	[31]
			NCT03217617	
Wiskott Aldrich syndrome	*WAS*	γ-retroviral Lentiviral (*ex-vivo*)	NCT01410825	[23]
			NCT01515462*; NCT01347242*; NCT01347346*; NCT03837483 NCT02333760	[32,33,44]
X-linked Chronic Granulomatous Disease	*CYBB*	γ-retroviral SIN γ-retroviral Lentiviral (*ex-vivo)* Adenoviral (*in-vivo*)	NCT00564759; NCT00927134*; NCT00394316; NCT00778882	[24,25,45]
			NCT01906541	[35]
			NCT01855685; NCT02234934*; NCT02757911; NCT03645486; NCT01381003	
			NCT06876363	
Lentiviral only				
P47^phox^ deficient Chronic Granulomatous Disease	*NCF1*	Lentiviral (*ex-vivo*)	NCT06253507; NCT05207657	[46]
IPEX Syndrome	*FOXP3*	Lentiviral (*ex-vivo*)	NCT05241444	[47]
Hemophagocytic lymphohistiocytosis	*UNC13D*	Lentiviral (*ex-vivo*)	NCT06736080	[48]
	*PRF1*	Lentiviral (*ex-vivo*)	Preclinical	[49]
Deficiency of Adenosine Deaminase 2	*ADA2*	Lentiviral (*ex-vivo*)	Preclinical	[50]
RAG2-SCID	*RAG2*	Lentiviral (*ex-vivo*)	Preclinical	[51]
X-linked lymphoproliferative disease	*SH2D1A*	Lentiviral (*ex-vivo*)	Preclinical	[52]
X-linked agammaglobulinemia	*BTK*	Lentiviral (*ex-vivo*)	Preclinical	[53]
AR-IFN-γR1 deficiency	*IFN-*γ*R1*	Lentiviral (*ex-vivo*)	Preclinical	[54]
RAG1-SCID	*RAG1*	Lentiviral (*ex-vivo*)	NCT04797260	[55]
Artemis-SCID	DCLRE1C	Lentiviral (*ex-vivo*)	NCT05071222; NCT03538899	[56]
Leukocyte adhesion deficiency	*ITGB2*	Lentiviral (*ex-vivo*)	NCT03812263*; NCT06282432; NCT03825783	[57,58]

Preclinical refers to experiments performed *in vitro* or in murine models only. * indicates a completed clinical trial. IEIs: Inborn errors of immunity; SIN: self-inactivating; WAS: Wiskott-Aldrich syndrome; IPEX: immunodysregulation, polyendocrinopathy, enteropathy, X-linked; ADA-SCID: adenosine deaminase-deficient severe combined immunodeficiency; *CYBB*: cytochrome b-245, beta chain; BTK: bruton tyrosine kinase.

**Table 2:** Overview of different base editors with their respective PAM sequences

	Editing window	PAM sequence preference	Reference
Adenine deaminase			
ABE8e NGG	4-8	NGG	[85]
ABE8eSPRY	4-8	NRN, NYN	[83]
ABE8e NG	4-8	NG	[85]
ABEmax NG	4-8	NG	[88]
ABEmax SPRY-GFP	4-8	NRN, NYN	[89]
Cytosine deaminase			
BE4max NRRH	4-8	NRRH	[90]
evoCDA BE4max NG	3-12	NG	[84]
evoFERNY BE4max NG	3-8	NG	[84]

NGG refers to the protospacer adjacent motif (PAM) recognised by wild-type Streptococcus pyogenes Cas9, where “N” represents any nucleotide followed by two guanines (“GG”). SPRY-GFP refers to an engineered base editor variant (e.g. ABE8e-SPRY-GFP) that contains mutations allowing recognition of a broader range of PAMs (such as NRN and NYN), with GFP typically included as a reporter or selection marker. NG is a relaxed PAM class (any nucleotide followed by guanine) recognised by certain engineered Cas9 variants (e.g. SpCas9-NG), enabling targeting beyond canonical NGG sites. NRN is a PAM motif where “N” is any nucleotide and “R” is a purine (adenine or guanine), used by some engineered Cas or base editor variants such as ABE8e-SPRY. NYN is another relaxed PAM motif where “Y” indicates a pyrimidine (cytosine or thymine), again enabling broader targetability by SPRY-derived editors. NRRH is a more specific PAM where “N” is any base, “R” is a purine (A or G), and “H” represents A, C, or T (i.e. any nucleotide except G); this is recognised by certain cytosine base editors such as BE4max NRRH. PAM: Protospacer adjacent motif; ABEs: adenine base editors.

**Table 3:** Gene editing strategies for IEIs and current trial status if applicable

Disease	Gene	Approach	Stage of development	Reference
HDR				
X-linked SCID	*IL2RG*	- HDR knock-in of full length *IL2RG* cDNA at translation start site using CRISPR/Cas & AAV6 in HSCs	Preclinical	[78]
X-linked hyper IgM syndrome	*CD40L*	- HDR knock-in of *CD40L* cDNA in 5’UTR with TALEN or CRISPR/Cas + AAV6 in HSCs & T cells - HDR knock-in of *CD40L* cDNA in 1st intron using CRISPR/Cas + AAV6 in HSCs and T cells	Preclinical Preclinical	[77] [94]
RAG1-SCID	*RAG1*	- HDR knock-in of *RAG1* cDNA in-frame in exon 2 using CRISPR/Cas + AAV6 or IDLV in HSCs	Preclinical	[95]
RAG2-SCID	*RAG2*	- HDR knock-in of *RAG2* cDNA upstream of START codon using CRISPR/Cas + AAV6 in iPSCs - HDR knock-in of *RAG2* cDNA upstream of START codon using CRISPR/Cas + AAV6 in HSCs - HDR knock-in of *RAG2* cDNA downstream of START codon using CRISPR/Cas + AAV6 in HSCs	Preclinical Preclinical Preclinical	[96] [78] [97]
Wiskott Aldrich syndrome	*WAS*	- HDR knock-in of *WAS* cDNA at translation start site using CRISPR/Cas + AAV6 in HSCs	Preclinical	[79]
X-linked agammaglobulinemia	*BTK*	- HDR knock-in of *Btk* cDNA + 300bp of terminal intron into exon 2 using CRISPR/Cas + AAV6 in HSCs	Preclinical	[98]
IL7Rα SCID	*IL7RA*	- HDR knock-in of promoterless *IL7RA* cDNA in exon 1 using CRISPR/Cas + AAV6 in T cells & HSCs	Preclinical	[99]
IPEX syndrome	*FOXP3*	- HDR knock-in of *FOXP3* cDNA at translation start site + tNGFR selectable marker using CRISPR/Cas + AAV6 in T cells & HSCs	Preclinical	[80]
X-linked chronic granulomatous disease	*CYBB*	- HDR knock-in of *CYBB* into AAVS1 safe harbour using ZFN + AAV6 in HSCs	Preclinical Preclinical	[100] [101]
		- HDR knock-in of *CYBB* at endogenous exon 2 using CRISPR/Cas + AAV6 in HSCs - HDR: Mutation-specific correction of c.676C > T using CRISPR/Cas + ssODN in HSCs	Preclinical	[81]
XMEN syndrome	*MAGT1*	- HDR knock-in of *MAGT1* in the endogenous exon 1 using CRISPR/Cas + AAV6 in HSCs & T cells	Preclinical	[102]
X-linked lymphoproliferative disease	*SH2D1A*	- HDR knock-in of *SH2D1A* cDNA within the first exon using TALEN + AAV6 or CRISPR/Cas + AAV6 in T cells	Preclinical	[103]
CTLA-4 Insufficiency	*CTLA4*	- HDR knock-in of *CTLA-4* cDNA in the first intron using CRISPR/Cas + AAV6 in T cells	Preclinical	[59]
Sting-associated vasculopathy with onset in infancy	*STING1*	- HDR knock-in of *coSTING1* cDNA (exon 5-8) downstream of endogenous intron 4 in patient-derived iPSCs and healthy-donor derived HSCs	Preclinical	[104]
Base editing				
X-linked SCID	*IL2RG*	- Base editing of the c.444C > T, p.Q144X mutation in *IL2RG*	Clinical	Not
			NCT06851767	published
CD35 SCID	*CD3D*	- Mutation specific correction of c.202C > T using adenine base editing in HSCs	Preclinical	[105]
X-linked chronic granulomatous disease		- Mutation specific correction of c.676C > T and c.1075G > A using adenine base editing in HSCs	Clinical NCT06325709	[106]
Prime editing				
P47^phox^ deficient chronic granulomatous disease	*NCF1*	- Prime editing to restore the 2 nucleotide GT deletion in HSCs	Clinical NCT06559176	[107]
Other				
Hemophagocytic lymphohistiocytosis	*UNC13D*	- CRISPR/Cas9-mediated excision of an intronic cryptic splice donor site using two gRNAs in murine HSCs	Preclinical	[108]
Severe congenital neutropenia	*ELANE*	- Disruption of *ELANE* promoter TATA box using dual CRISPR-Cas9D10A nickases operating on opposite strands	Preclinical	[109]

Preclinical refers to experiments performed *in vitro* or in murine models only. * indicates a completed clinical trial. START refers to the translation start site of a gene, typically the ATG codon where protein synthesis begins. iPSCs: induced pluripotent stem cells, are somatic cells that have been reprogrammed back into a pluripotent state, enabling them to differentiate into multiple lineages. TATA refers to the TATA box, a core promoter DNA sequence (usually TATAAA) that helps position RNA polymerase II for transcription initiation. BTK: Bruton tyrosine kinase is a cytoplasmic tyrosine kinase essential for B-cell development; mutations in BTK cause X-linked agammaglobulinemia. tNGFR: truncated nerve growth factor receptor is a selectable surface marker often used in gene therapy constructs to identify or enrich modified cells. CYBB: Cytochrome b-245, beta chain encodes gp91^phox^, a subunit of the phagocyte NADPH oxidase complex; mutations result in X-linked chronic granulomatous disease. XMEN: X-linked immunodeficiency with Magnesium defect, Epstein–Barr virus infection, and Neoplasia is caused by MAGT1 mutations and leads to T-cell dysfunction, chronic EBV infection, and lymphoma risk. SCID: severe combined immunodeficiency refers to a group of disorders characterised by profound defects in T-cell and, in many cases, B-cell and NK-cell function. ELANE refers to the gene encoding neutrophil elastase; mutations in ELANE are a common cause of severe congenital neutropenia. IEIs: Inborn errors of immunity; HDR: homology-directed repair; CRISPR: clustered regularly interspaced short palindromic repeats; cDNA: complementary DNA; HSC: haematopoietic stem cell; IDLVs: integrase-deficient lentiviruses; AAV6: adeno-associated virus type 6; WAS: Wiskott-Aldrich syndrome; IPEX: immunodysregulation, polyendocrinopathy, enteropathy, X-linked; ZFNs: zinc-finger nucleases; ssODN: single-stranded oligodeoxynucleotides; GT: gene therapies; gRNA: guide RNA.

## Data Availability

Not applicable.
